# Evaluating the association of osteoporosis with inhaled corticosteroid use in chronic obstructive pulmonary disease in Taiwan

**DOI:** 10.1038/s41598-020-80815-y

**Published:** 2021-01-12

**Authors:** Kai-Lin Chiu, Chun-Chen Lee, Chung-Yu Chen

**Affiliations:** 1 Master Program in Clinical Pharmacy, School of Pharmacy, Kaohsiung Medical University, No. 100, Shihcyuan 1st Rd., Sanmin District, Kaohsiung, 80708 Taiwan, ROC; 2 Department of Pharmacy, Kaohsiung Medical University Hospital, No. 100, Tzyou 1st Rd., Sanmin District, Kaohsiung, 80708 Taiwan, ROC; 3 Department of Medical Research, Kaohsiung Medical University Hospital, Kaohsiung, Taiwan; 4 Center for Big Data Research, Kaohsiung Medical University, Kaohsiung, Taiwan

**Keywords:** Chronic obstructive pulmonary disease, Epidemiology

## Abstract

Chronic obstructive pulmonary disease (COPD) is characterized by airflow limitation and osteoporosis is the major comorbidity associated with poor prognosis in COPD. However, the effect of inhaled corticosteroids (ICS) on bone mineral density among COPD remains uncertain. There is the urgent need to examine whether the long-term ICS use may increase the risk of osteoporosis. In this nested case–control study retrieved from the Taiwan National Health Insurance Research Database from 2002 to 2017, the study aimed to investigate risk of osteoporosis associated with ICS, focusing on the dosage and duration of ICS therapy. Cases with osteoporosis or osteoporotic fractures claims were defined and matched to 3 randomly selected controls. Conditional logistic regressions were used to estimate odds ratios of osteoporosis from ICS treatment measured in 3 years before the index date. This population-based study included 891,395 patients with COPD, where after matching had 58,048 case groups and 174,144 matched control groups. After adjusting for potential confounders, ICS use in COPD was associated with a 1.053-fold (95% confidence interval 1.020–1.087) increased osteoporosis risk, where 7892 (13.59%) ICS use in case and 22,580 (12.97%) in control. New ICS use in COPD patients is associated with increased osteoporosis risk, regardless of exposure period.

## Introduction

Chronic obstructive pulmonary disease (COPD) is characterized by progressive airflow limitation and is associated with chronic inflammatory response in the lungs and airways^1^. The symptoms of COPD is shortness of breath, chronic cough with mucus, and limited daily activities^2,3^. COPD had linked with many comorbidities and was the third leading cause of death in World Health Organization (WHO) in 2016^4^. Osteoporosis is one of the major comorbidity, and associated with poor health status and prognosis in COPD. Various risk factors may contribute to the disease prognosis and have a major impact on health status and survival^5^.

Inhaled corticosteroids (ICS) has been considered the standard therapy for COPD to reduce exacerbation risks and inflammation from the Global Initiative for Chronic Obstructive Lung Disease (GOLD) 2020^6^. However, the impact of ICS on fracture and osteoporosis had yielded the conflicting conclusions^7–14^. Inhaled triamcinolone use for 3 years was associated with bone mass reduction in the Lung Health Study II^15^. Inhaled budesonide in the EUROSCOP trial or inhaled fluticasone use for 3 years in the TORCH trial had not showed bone mass reduction^10,14^. These randomized clinical trials (RCT) had found that no increasing the osteoporosis risk, but slight increasing in high-dose ICS^10–15^. However, the follow-up time was not long enough and not all ICS drug was used in these studies, so all of these limitations could weaken the causality and generalizability.

The present studies that investigating the effect of ICS on osteoporosis are still controversial. We used the Taiwan’s National Health Insurance Research database (NHIRD) to conduct the nested case–control study. This study was to investigate risk of osteoporosis associated with ICS in a nationwide population of patients with COPD, focusing on the dosage and duration of ICS therapy.

## Results

### Study population

**Figure 1:**
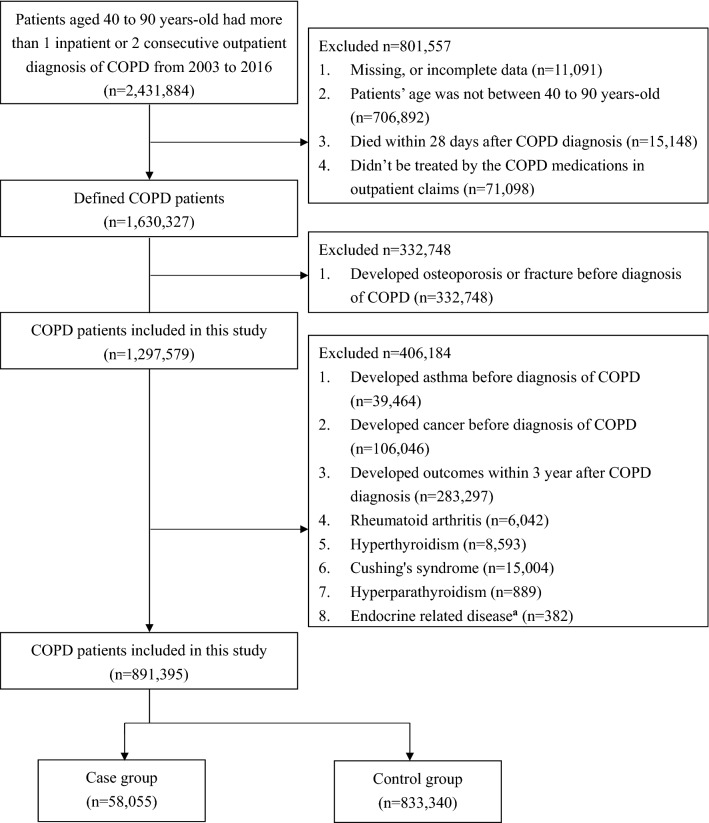
Study flow diagram of study cohort selection. Endocrine related disease: growth hormone deficiency, hypogonadism, hypophosphatasia, and porphyria.

The COPD cohort included 891,395 patients, which had 58,055 case group and 833,340 control group (Fig. 1). After 1-to-3 matching, case group had 58,048 patients with the mean age of 66.23 years, and control group had 174,144 patients with the mean age of 66.32 years. In case group, there were 29,611 osteoporosis and 28,437 osteoporotic fractures. Then, the male was 51.24% in case and control group. The age group, gender, COPD severity, asthma, dyslipidemia, hypertension, diabetes mellitus, chronic kidney disease, chronic liver disease, and malignancy as matching factors were balanced between two groups. The baseline characteristics before and after matching were listed in Table 1.

**Table 1:** Baseline characteristic between cases and matched controls.

Baseline characteristics N (%)	At baseline^a^			During follow-up^b^		
	Case (N = 58,048)	Control (N = 174,144)	*p-*value	Case (N = 58,048)	Control (N = 174,144)	*p-*value
**Probability mean (SD)**	0.2001 (0.0047)	0.2001 (0.0047)	1.000	0.2001 (0.0047)	0.2001 (0.0047)	1.000
**Age mean (SD)**	66.23 (11.57)	66.32 (11.45)	0.1235	66.23 (11.57)	66.32 (11.45)	0.1235
**Age group**			0.1386			0.1386
40 ≤ age < 50	6057 (10.43)	17,053 (9.79)		6057 (10.43)	17,053 (9.79)	
50 ≤ age < 60	10,649 (18.35)	32,299 (18.55)		10,649 (18.35)	32,299 (18.55)	
60 ≤ age < 70	15,267 (26.30)	47,228 (27.12)		15,267 (26.30)	47,228 (27.12)	
70 ≤ age < 80	19,224 (33.12)	56,796 (32.61)		19,224 (33.12)	56,796 (32.61)	
80 ≤ age	6851 (11.80)	20,768 (11.93)		6851 (11.80)	20,768 (11.93)	
**Sex**			1.000			1.000
Male	29,742 (51.24)	89,226 (51.24)		29,742 (51.24)	89,226 (51.24)	
Female	28,306 (48.76)	84,918 (48.76)		28,306 (48.76)	84,918 (48.76)	
**Insurance premium (NT$) **^**c**^			< 0.0001			< 0.0001
≤ Monthly minimum wage	14,115 (24.32)	44,301 (25.44)		14,115 (24.32)	44,301 (25.44)	
> Monthly minimum wage	43,933 (75.68)	129,843 (74.56)		43,933 (75.68)	129,843 (74.56)	
**Urbanization level**			< 0.0001			< 0.0001
Urban	27,994 (48.23)	87,237 (50.09)		27,994 (48.23)	87,237 (50.09)	
Suburban	23,346 (40.22)	68,091 (39.10)		23,346 (40.22)	68,091 (39.10)	
Rural	6708 (11.56)	18,816 (10.80)		6708 (11.56)	18,816 (10.80)	
**Comorbidity**						
Asthma	0 (0.00)	0 (0.00)	NA	8270 (14.25)	24,646 (14.15)	0.5732
Dyslipidemia	636 (1.1)	1918 (1.10)	0.9085	14,965 (25.78)	44,438 (25.52)	0.2095
Hypertension	4698 (8.09)	15,298 (8.78)	< 0.0001	36,462 (62.81)	109,684 (62.98)	0.4597
Diabetes mellitus	2,605 (4.49)	8012 (4.60)	0.2585	17,605 (30.33)	52,835 (30.34)	0.9584
Chronic kidney disease	347 (0.60)	1100 (0.63)	0.3690	4870 (8.39)	14,610 (8.39)	1.000
Chronic liver disease	916 (1.58)	2237 (1.28)	< 0.0001	7640 (13.16)	23,036 (13.23)	0.6815
Malignancy	0 (0.00)	0 (0.00)	NA	11,465 (30.56)	34,460 (19.79)	0.8450
Acute bronchitis	1195 (2.06)	3445 (1.98)	0.2307	17,740 (30.56)	51,333 (29.48)	< 0.0001
Pneumonia	1835 (3.16)	6285 (3.61)	< 0.0001	8255 (14.22)	29,406 (16.89)	< 0.0001
Alcohol-related disease	192 (0.33)	259 (0.15)	< 0.0001	468 (0.81)	527 (0.30)	< 0.0001
Renal failure	116 (0.20)	354 (0.20)	0.8729	1724 (2.97)	5168 (2.97)	0.9775
Dementia	103 (0.18)	728 (0.42)	< 0.0001	4688 (8.08)	12,846 (7.38)	< 0.0001
Alzheimer's disease	6 (0.01)	68 (0.04)	0.0008	481 (0.83)	1009 (0.58)	< 0.0001
Depression	245 (0.42)	504 (0.29)	< 0.0001	4163 (7.17)	8521 (4.89)	< 0.0001
**ASCVD**						
Coronary artery disease	1940 (3.34)	6012 (3.45)	0.2059	13,805 (23.78)	40,675 (23.36)	0.0364
Peripheral vascular disease	142 (0.24)	489 (0.28)	0.1471	3288 (5.66)	8725 (5.01)	< 0.0001
Ischemic stroke/transient ischemic attack	1249 (2.15)	4575 (2.63)	< 0.0001	9306 (16.03)	25,712 (14.76)	< 0.0001
Hemorrhagic stroke	223 (0.38)	1036 (0.59)	< 0.0001	1132 (1.95)	3512 (2.02)	0.3208
Heart failure	744 (1.28)	2683 (1.54)	< 0.0001	7402 (12.75)	22,477 (12.91)	0.3322
Left ventricular hypertrophy	57 (0.10)	176 (0.10)	0.8499	510 (0.88)	1171 (0.67)	< 0.0001
Atrial fibrillation	880 (1.52)	2943 (1.69)	0.0043	7171 (12.35)	22,397 (12.86)	0.0015
**Medication for COPD**						
LABA	1565 (2.70)	4673 (2.68)	0.8705	1106 (1.91)	2991 (1.72)	0.0029
LABA/ICS	3198 (5.51)	9199 (5.25)	0.0353	4670 (8.05)	12,462 (7.16)	< 0.0001
LAMA	781 (1.35)	2262 (1.30)	0.3935	2131 (3.67)	4852 (2.79)	< 0.0001
LABA/LAMA	2302 (3.97)	6798 (3.90)	0.5049	3501 (6.03)	8292 (4.76)	< 0.0001
SABA	7321 (12.61)	22,143 (12.72)	0.5170	8378 (14.43)	26,584 (15.27)	< 0.0001
SAMA	4741 (8.17)	14,243 (8.18)	0.9303	5419 (9.34)	18,286 (10.50)	< 0.0001
SABA/SAMA	2306 (3.97)	6967 (4.00)	0.7643	5862 (9.37)	18,128 (10.41)	0.0329
Systemic beta-2-adrenoreceptor agonists	30,300 (52.20)	89,561 (51.43)	0.0013	19,263 (33.18)	57,316 (32.91)	0.2280
ICS	1955 (3.37)	5896 (3.39)	0.8372	1328 (2.29)	4079 (2.34)	0.4504
Corticosteroid	22,291 (38.40)	63,952 (36.72)	< 0.0001	26,496 (45.64)	68,365 (39.26)	< 0.0001
Methyl-xanthines	37,823 (65.16)	110,958 (63.72)	< 0.0001	24,232 (41.74)	70,919 (40.72)	< 0.0001
Antibiotic	32,043 (55.20)	94,108 (54.04)	< 0.0001	42,124 (72.57)	88,393 (50.76)	< 0.0001
**COPD severity**						
Moderate exacerbations			0.4123			0.9563
0	46,980 (80.93)	141,345 (81.17)		43,990 (75.78)	131,877 (75.73)	
1	5247 (9.04)	15,462 (8.88)		6607 (11.38)	19,895 (11.42)	
≥ 2	5821 (10.03)	17,337 (9.96)		7451 (12.84)	22,372 (12.85)	
Severe exacerbations			0.5176			0.1101
0	53,907 (92.87)	161,491 (92.73)		49,934 (86.02)	150,404 (86.37)	
1	3367 (5.80)	10,326 (5.93)		4531 (7.81)	13,277 (7.62)	
≥ 2	774 (1.33)	2327 (1.34)		3583 (6.17)	10,463 (6.01)	
COPD exacerbations risk			0.9106			0.1624
Low	49,617 (85.48)	148,884 (85.49)		46,217 (79.62)	139,119 (79.89)	
High	8431 (14.52)	25,260 (14.51)		11,831 (20.38)	35,025 (20.11)	
**Co-medication**^**d**^						
SSRI	1400 (2.41)	3663 (2.10)	< 0.0001	2701 (4.65)	5233 (3.00)	< 0.0001
Antiepileptic drug	16,757 (28.37)	46,201 (26.53)	< 0.0001	21,387 (36.84)	56,333 (32.35)	< 0.0001
Chemotherapy	208 (0.36)	676 (0.39)	0.3117	1036 (1.78)	3910 (2.25)	< 0.0001
GnRH agonist	15 (0.03)	55 (0.03)	0.4901	209 (0.36)	440 (0.25)	< 0.0001
Aromatase inhibitors	11 (0.02)	49 (0.03)	0.2330	111 (0.19)	324 (0.19)	0.8031
Lithium	74 (0.13)	149 (0.09)	0.0047	72 (0.12)	119 (0.07)	< 0.0001
Proton pump inhibitors	3639 (6.27)	10,207 (5.76)	0.0003	6200 (10.68)	19,157 (11.00)	0.0324
Thiazolidinediones	1186 (2.04)	3151 (1.81)	0.0003	648 (1.12)	1637 (0.94)	0.0002
Thyroid hormone	423 (0.73)	1484 (0.85)	0.0043	838 (1.44)	2886 (1.66)	0.0004
Heparin	132 (0.23)	402 (0.23)	0.8807	477 (0.82)	2264 (1.13)	< 0.0001
Immunosuppressants	29 (0.05)	62 (0.13)	0.1302	67 (0.12)	167 (0.10)	0.1992
ADT	15 (0.03)	55 (0.03)	0.4901	212 (0.37)	444 (0.25)	< 0.0001
Aluminum	24,130 (41.57)	65,952 (37.87)	< 0.0001	10,301 (17.75)	26,874 (15.43)	< 0.0001
LMWH	40 (0.07)	137 (0.08)	0.4605	95 (0.16)	468 (0.27)	< 0.0001
Warfarin	671 (1.16)	2122 (1.22)	0.2309	1254 (2.16)	3638 (2.09)	0.3009

*LABA* long-acting beta-agonist, *ICS* inhaled corticosteroids, *LAMA* long-acting muscarinic antagonists, *SABA* short-acting beta-agonist, *SAMA* short-acting muscarinic antagonists, *SSRI* selective serotonin reuptake inhibitors, *GnRH agonist* gonadotropin-releasing hormone agents, *ADT* androgen deprivation therapy, *Immunosuppressants* cyclosporine or tacrolimus, *LMWH* low-molecular-weight heparin, *NA* not applicable.

^a^All comorbidities were measured in the year preceding entry date. COPD severity, medication for COPD, and co-medication were measured in the year after entry date.

^b^All comorbidities, COPD severity, medication for COPD, and co-medication were measured in the year before index date.

^c^The range of minimum incomes from the government announcements.

^d^The duration of co-medication was more than 30 day.

### Risk of osteoporosis with ICS use

As showed in Table 2, COPD patients with ICS use were associated with increasing osteoporosis risk based on different recency of therapy, especially in the past ICS use (2–3 year). During the 3-year observation period, the study consisted of 30,472 (13.12%) ICS use in 232,192 COPD population, where 7892 (13.59%) ICS use in case group and 22,580 (12.97%) in control group. COPD population with ICS use had 1.053 fold-risk for osteoporosis (p-value = 0.0013). Among past users, ICS treatment was associated 1.09-fold (p-value < 0.0001) increased osteoporosis risk. Then recent ICS users had no statistically significant difference in risk of osteoporosis (p-value = 0.2751). While current ICS users had 2.9% reduction osteoporosis risk without statistically significant difference (p-value = 0.3019).

**Table 2:** Risk of osteoporosis with use of ICS compared with nonuse, stratified by exposure time.

3 year	Case	Control^a^	Crude OR (95% CI)	P value	Adjusted OR^b^ (95% CI)	P value
ICS exposure, no (%)	N = 58,048	N = 174,144				
No use of ICS	50,156 (86.4)	151,564 (87.03)	1 (reference)		1 (reference)	
ICS use	7892 (13.6)	22,580 (12.97)	1.056 (1.028, 1.086)	< 0.0001	1.053 (1.020, 1.087)	0.0013
Current use (< 1 year)	1871 (3.2)	5838 (3.4)	0.969 (0.919, 1.022)	0.2414	0.971 (0.918, 1.027)	0.3019
Recent use (1–2 year)	1789 (3.1)	5202 (3.0)	1.040 (0.984, 1.098)	0.1635	1.032 (0.975, 1.092)	0.2751
Past use (2–3 year)	4232 (7.3)	11,540 (6.6)	1.108 (1.068, 1.150)	< 0.0001	1.090 (1.048, 1.135)	< 0.0001

*OR* odds ratio.

^a^Case and control matched by age group, gender, COPD severity, asthma, dyslipidemia, hypertension, diabetes mellitus, chronic kidney disease, chronic liver disease, and malignancy.

^b^Adjusted for age group, gender, urbanization level, income, COPD severity, comorbidity, the oral steroids use and co-medication.

Furthermore, the osteoporosis risk between dose and exposure time of ICS were also evaluated in Table 3. First, while adjusting for other effects, COPD patients with high-dose ICS use had 1.085 fold-risk for osteoporosis (p-value < 0.0001), compared to no ICS use. It was showed that ICS significantly increasing chance of osteoporosis until over high-dose ICS use. Second, low-dose ICS exposure had 0.714 fold-risk for osteoporosis (p-value < 0.0001), which indicated decreasing hazard of osteoporosis than no ICS exposure. Last, COPD patients in high adherence group was 1.320 fold-risk for osteoporosis (p-value < 0.0001), in low adherence group was 1.040 fold-risk for osteoporosis (p-value = 0.0151) when adjusting other covariates. It was reflected that the longer exposure period, the higher osteoporosis chance. All in all, it was showed that ICS could increase the risk of osteoporosis, when COPD population are with high dose ICS use or in high adherence group in 3-year exposure period.

**Table 3:** Risk of osteoporosis with use of ICS, stratified by the mean daily dose, and MPR of ICS.

3 years	Case	Control^a^	Crude OR (95% CI)	P value	Adjusted OR^b^ (95% CI)	P value
ICS exposure, no (%)	N = 58,048	N = 174,144				
**The mean daily dose**						
No use of ICS	50,156 (86.4)	151,564 (87.03)	1 (reference)		1 (reference)	
ICS use	7892 (13.6)	22,580 (12.97)	1.056 (1.028, 1.086)	< 0.0001	1.053 (1.020, 1.087)	0.0013
Low dose	266 (0.46)	1146 (0.66)	0.702 (0.614, 0.802)	< 0.0001	0.714 (0.623, 0.818)	< 0.0001
Median dose	755 (1.3)	2388 (1.37)	0.956 (0.880, 1.038)	0.2840	0.96 (0.882, 1.044)	0.3363
High dose	6871( 11.84)	19,046 (10.94)	1.091 (1.059, 1.123)	< 0.0001	1.085 (1.049, 1.121)	< 0.0001
**MPR**						
No use of ICS	50,156 (86.4)	151,564 (87.03)	1 (reference)		1 (reference)	
ICS use	7892 (13.6)	22,580 (12.97)	1.056 (1.028, 1.086)	< 0.0001	1.053 (1.020, 1.087)	0.0013
High adherence group (MPR ≥ 0.8)	7448 (12.83)	21,561 (12.38)	1.044 (1.015, 1.074)	0.0027	1.040 (1.008, 1.074)	0.0151
Low adherence group (MPR < 0.8)	444 (0.76)	1019 (0.59)	1.318 (1.178, 1.474)	< 0.0001	1.320 (1.177, 1.480)	< 0.0001

*OR* odds ratio.

^a^Case and control matched by age group, gender, COPD severity, asthma, dyslipidemia, hypertension, diabetes mellitus, chronic kidney disease, chronic liver disease, and malignancy.

^b^Adjusted for age group, gender, urbanization level, income, COPD severity, comorbidity, the oral steroids use and co-medication.

### Sensitivity and subgroup analysis

**Figure 2:**
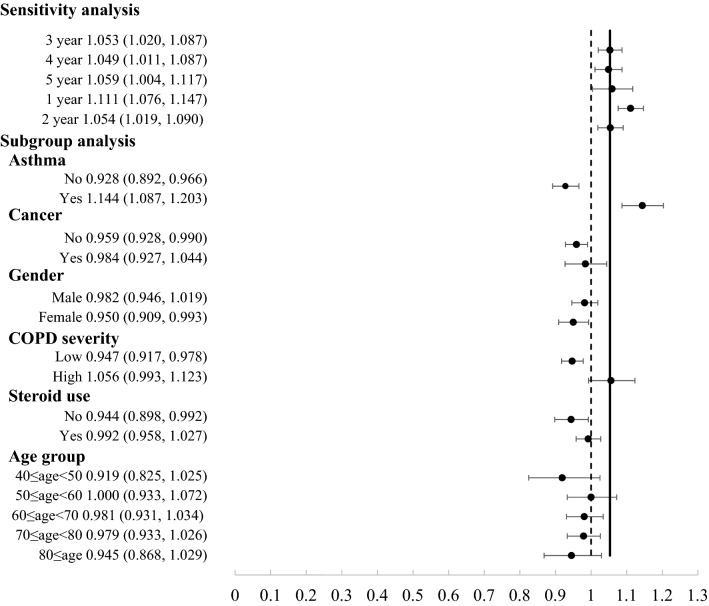
Sensitivity analysis and subgroup analysis of osteoporosis risk with ICS use. We calculated the sensitivity analysis and subgroup analysis for the risk of osteoporosis with ICS use, stratified by the potential factors.

The subgroup analysis and sensitivity analysis were showed in Fig. 2. Most of the sensitivity analyses indicated robustness of the main findings. COPD population with ICS use had the chance of increasing osteoporosis risk with statistical significance difference regardless the exposure period. The adjusted ORs ranged from 1.049 to 1.111 across the different exposure period. Nevertheless, we extended or shorted the exposure period, the trend that ICS use could increase the risk of osteoporosis remained robust. There were 6 potential confounders as follow: prior asthma, prior cancer, gender, age group, COPD severity, and the oral steroid use. Subgroup analysis revealed that this effect between ICS on osteoporosis and potential confounders. Furthermore, cigarette smoking increased one percentage exposure was 1.006 fold-risk for osteoporosis (p-value < 0.0001). The covariates including in adjusted model were selected as different methods (eTable 2 in the Supplement). The trend that ICS use was associated with osteoporosis remained constant. Whether the balance factors were included or not, it would not change the outcome.

After many different analysis were conducted to reduce bias, the main findings that COPD patients with ICS use was associated the osteoporosis risk was robust and constant. And ICS use had the dose-dependence and duration-dependence associations with osteoporosis risk in 3-year exposure period.

## Discussion

Our large observational study of more than 890,000 patients with COPD showed an approximate 1.053-fold increased risk of osteoporosis with ICS therapy, but the risk was even absent, with current (< 1 year) and recent use (1–2 year) of ICS. This finding was replicated in the different selection of ICS prescription records to approve the long-term effect. As a whole, we had provided the first evidence to indicate that long-term high-dose ICS use was associated with the risk of osteoporosis in patients with COPD and low dose ICS use had a significantly decreased osteoporosis risk.

Although ICS could reduce exacerbation, the impact on bone mineral density was controversial and still needed to be considered^16^. Previous RCT studies^10,11,15^ that taken bone mineral density as definition and not included all kind of ICS, and those results showed that the bone mineral density was slightly decreased with almost 3-year ICS use. Then, the related case–control studies that used fracture as outcome, and reported that COPD with high-dose ICS use was associated with an increased fracture risk^17,18^. In summary, the outcome definition was not similar, and risk of osteoporosis with ICS use was still controversial. Currently, none of study examined the association between ICS use and osteoporosis. Therefore, the ability to observe risk of osteoporosis with ICS in our study including a nationwide population of patients with COPD. On the whole, COPD patients with ICS use had increased the risk of osteoporosis in our study.

The phenomenon that COPD patients with 3-year ICS use may increase risk of osteoporosis was approved. In our study, dose-dependent effect and time-dependent effect were also showed. The data about the ICS potency was not enough. Hence, the ICS dose was converted to low, median, and high mean daily dose from GINA^19^. First, COPD with high dose ICS use had the 8.5% chance of increasing osteoporosis (aOR = 1.085; p-value < 0.0001). It was showed that high dose ICS had the effect on increasing osteoporosis risk. This trend was similar with the previous studies^17,18^. Furthermore, the results about low ICS dose in our study had the same trend with other related studies^17,18,20^. As previous study, low dose oral steroids did not showed increasing fracture risk, and loss of bone mineral density occurred rapidly, but can be reversed^21^. Otherwise, using low dose ICS patients may more health than high dose population, which increasing daily activities also increased bone mineral density. Therefore, from our results, low dose ICS use had a significantly decreased osteoporosis risk than non-users or high dose ICS. The MPR was conducted to reveal the time-dependent effect. COPD in high adherence group was more 32% chance to have osteoporosis (p-value < 0.0001). It was showed that day of ICS use was associated with osteoporosis. All in all, not only the association between ICS and osteoporosis was showed, but also the dose-dependent effect and time-dependent effect were revealed in our study. The dose-dependent effect and time-dependent effect of ICS had been reported in lot of studies in different study design and make the results more robust. In summary, COPD patients with ICS use had chance to increase osteoporosis risk with dose-dependent effect and time-dependent effect.

In subgroup analysis, the results revealed that this effect of ICS on osteoporosis under different scenarios. In scenarios of prior asthma and COPD severity, it was showed that the risk may be influenced by these two factors. Nevertheless, in other situation, the chance between ICS and osteoporosis risk was reduced. These results reflected that the proportion of ICS use in case group was less than in control group after classified into these factors. In summary, we could understand the risk of osteoporosis with ICS use in different situation.

In sensitivity analysis, the exposure period was changed to reveal the robustness of the main findings. The exposure period included 1-year, 2-year, 4-year, and 5-year prior index date. Regardless of the exposure period, the trend that COPD patients with ICS use had the chance to increase the osteoporosis risk was robustness. None of previous study took the same study design as our study. The related study that the use of triamcinolone was associated with loss of BMD after 3 years of treatment^13^. Until now, there were some related studies that used clinical examination, not the diagnosis code as outcome definition on this topic. The comparability of the related studies was not enough. In conclusion, our finding kept solid after the subgroup and sensitivity analysis were conducted.

The potential mechanisms have been proposed to interpret the osteoporosis risk increasing from ICS use. The preferred ICS administration for COPD was inhalation, which delivered the drug direct to the lung, that can act at local and minimized the systemic effect, but some proportion of drug was still delivered to the systemic circulation with unchanged form that potentially causing extra-pulmonary side effects. However, if the drug was not decomposition by month, the left drug may be swallowed and absorbed from gastrointestinal tract. Then the ICS entered the systemic circulation and caused systemic side effects^22^.

To our knowledge, our study is the first population-based nested case–control study to evaluate the risk of osteoporosis with ICS use in COPD patients. This study have many important strengths. The first strength is that we conducted this study by using large population database. The 99.9% of 23 million Taiwan’s population and the 93.03% of the healthcare providers were covered by the NHIRD. Therefore, this study contains the large sample size and has the national representative. The sample size is big enough to provide enough power of the statistical analysis in our study. The second strength is long observation time. The study has 15-year period to follow. Then, the osteoporosis is the chronic disease, so the exposure time is long enough to observe the outcome development. Third, the study design is strength. The nested case–control study design could reduce the misclassification of ICS use. And the ICS exposure could be calculated within the period to confirm the time-dependent effect. Hence, our study could reflect the real world data in clinical situation compared the RCT. Those RCT did not included all ICS and the populations were restricted. The COPD population in our study were more similar to the real world. Further, the results are robust across several different definition of ICS use and exposure period.

The study has several limitations. The study population and all outcome definitions are based on ICD-9 CM and ICD-10 CM, not clinical diagnosis because the examination results and lab data are lack in NHIRD. Despite, we use many alternative definitions that combined diagnosis code with medication or examination to improve accuracy, and the proportion of comorbidities in our study was similar with other Taiwan database studies^23,24^. The comorbidities in our study were in line with the clinical conditions. The limitation of NHIRD leads to lack of smoking status, body mass index, life style, lung function, and bone mineral density. Despite the smoking status as adjusted factor in sensitivity analysis from the National Health Interview Survey, not direct from the study population from NHI, the ecological bias may appear and the percentage cannot indicated individual smoke status hence the confounding bias is likely. The self-pay drug did not be included in NHIRD and could not be analyzed. Then the other important factors could not be adjusted and may influence the outcome. Nerveless, the diagnosis of osteoporosis may be underdiagnosed, but the risk of osteoporosis with ICS use is still showed in this situation. Finally, building the propensity score on all factors would lead to more than half of population lost in study population, which causing the population representative would loss and the statistical power would be loss in our study. Therefore, we chose more important factors (age group, gender, COPD severity, asthma, dyslipidemia, hypertension, diabetes mellitus, chronic kidney disease, chronic liver disease, and malignancy) as matching factors.

In conclusion, the finding of our study suggested that ICS use in COPD patients had the chance to increase osteoporosis risk. And ICS use had the dose–response and duration-response associations with osteoporosis risk in 3-year exposure period. The main finding remained solid and consistent after the sensitivity analysis. In summary, the ICS use had the chance to increase the osteoporosis risk among COPD patients. Under this circumstances, COPD patients with ICS use had to pay attention to the osteoporosis occurrence.

## Methods

### Study design and data source

We performed the national-databased and population-based nested case–control study of COPD population aged more than 40 years or older. Taiwan established the National Health Insurance (NHI) program since 1995 which covers the 99.9% of 23 million Taiwan’s population and the 93.03% of the healthcare providers^25^. The study was conducted from the Taiwan National Health Insurance Research Database (NHIRD) from January 1, 2002, to December 31, 2017, which contained all medical claims, pharmacy claims and multiple cause of death datasets records. We independently conducted this study at a subcenter of the Health and Welfare Data Science Centers at Kaohsiung Medical University. This study was approved by the institutional review board of Kaohsiung Medical University Chung-Ho Memorial Hospital (KMUHIRB-EXEMPT(I)-20180043). This study was performed in accordance with the ethical standards established in the 1964 Declaration of Helsinki and its later amendments. This study conducted by NHIRD and all the individual information data are de-identified, we could not obtain informed consent from study population.

### Identification of study cohort

We identified newly diagnosed COPD patients with more than 2 outpatient or an inpatient visit records from January 1, 2003 to December 31, 2016 (*International Classification of Diseases, Ninth Revision* [ICD-9] codes 490, 491, 492, 496 and *Tenth Revision* [ICD-10] codes J40, J41, J43, J44). Patients who were 40 years or older and were accompanied by outpatient records of using COPD medications within 1 year after the primary COPD diagnosis date were defined as COPD patients. The cohort entry date was defined as the primary COPD diagnosis date. We excluded patients who had osteoporosis, fracture, asthma, or cancer before diagnosis of COPD (Fig. 1). The study cohort was followed up until the earliest osteoporosis or osteoporotic fractures outcome (defined in the case identification), death, NHIRD withdrawal, or the end of this study (December 31, 2017), whichever occurred first.

### Case identification

We identified cases as patients who had more than an inpatient or 2 outpatient diagnosis with osteoporosis or osteoporotic fractures (ICD-9 code 733, 805.2–805.9, 820, 812, 813 and ICD-10 code M80-M82, S21, S22, S32, S72, S79, S42, S49, S52, S59) between January, 1, 2004 and December, 31, 2017. The first osteoporosis or osteoporotic fracture date was index date.

For control group selection, each case was matched to three randomly selected control by age group, gender, COPD severity, asthma, dyslipidemia, hypertension, diabetes mellitus, chronic kidney disease, chronic liver disease, and malignancy, from the pool of patients who didn’t have outcome by cohort entry date (± 180 days). After the matching, the outcome date of case group was assigned as the index date to control group for case and control groups with the same probability to occur of osteoporosis outcome during follow-up.

### Exposure measurement

For the ICS exposure definition, we examined the 3 years ICS records before the index date for both case and control groups. Then, the drug use was based on the prescription date, duration, and dose of ICS prescriptions in the 3-years period. The duration was classified into three groups, current (≤ 1 year), recent (1–2 year), recent past (2–3 year), based on the first prescription date preceding the index date in 3 years (in eFigure 1 in the Supplement). The mean daily dose was then recoded into three group as follows: low, median, high dose. The ICS dose exposure will be categorized from GINA recommendation^19^.

### Measurement of covariates

Several covariates, such as prior asthma, malignancy, rheumatoid arthritis, Cushing's syndrome, hyperthyroidism, and use of systemic corticosteroids, were reported as potential confounders of osteoporosis and used as the exclusion criteria^26^. We use the proxy indicators to confirm COPD severity, including the number of COPD-related outpatient, hospital, or emergency room (ER) records and the drug use of short acting beta-agonist (SABA), respiratory antibiotics, or oral corticosteroid (in eTable 3 in the Supplement)^6,23^. The severe exacerbations mean exacerbation leading to ER or a hospital records. Instead, the moderate exacerbations mean not leading to hospital records but patients were treated with SABA plus antibiotics or oral corticosteroid. Other factors related to ICS use or osteoporosis were considered (in eTable 1 in the Supplement). We first measured these factors in the year prior index date, such as dyslipidemia, diabetes mellitus, hypertension, chronic kidney disease, and chronic liver disease. Second, we included these confounders as the matching factors in a logistic regression model to estimate the probability of encountering an osteoporosis outcome during follow-up.

### Statistical analysis

In baseline characteristics, continuous variables were presented as means (standard deviation) and categorical variables were presented as the percentage. Continuous variables were analyzed by Student’s T-test and categorical variables were analyzed by Chi-square test. We used conditional logistic regression to estimate the odds ratio (OR) of osteoporosis with ICS use. The conditional logistic regression was adjusted with all covariates were measured in 1 year before the index date, including age group, gender, urbanization level, income, COPD severity, comorbidity, the oral steroids use and co-medication. Data processing and statistical analysis were performed with the use of SAS 9.4. Software. The statistical significance was determined at two-tailed and *P* < 0.05.

### Sensitivity and subgroup analysis

Several additional analyses were conducted to address the potential confounders. First, we stratified the population who had used the oral prednisolone to avoid the effect of oral steroids. The impact of oral steroids on osteoporosis were approved the positive association. Second, we stratified analyses by age group, gender, asthma, cancer, use of oral steroids, and COPD severity to estimate the association in different population. Third, to determine the time-dependent relationship between ICS and osteoporosis, we classified the first and recent date of ICS use in 3 years before the index date. Forth, the dose of ICS was calculated to determine dose-dependent relationship. The mean daily ICS dose was classified into three dose categories, including low, median, and high dose^27^. Fifth, the duration of ICS use was calculated as the measuring medication adherence (MPR). MPR was the total day of ICS use in the exposure period, which had two group, high adherence group (MPR ≥ 0.8) and low adherence group (MPR < 0.8). Sixth, we also repeated the analyses within ICS group to determine time-dependent relationship, by changing the length of exposure period in sensitivity analysis. Finally, in sensitivity analysis, cigarette smoking will be calculated from the National Health Interview Survey, The information of smoking exposure percentage were presented as a percentage of the population of one county or city and the percentage as adjusted factor in study model. We used daily smoking percentage in National Health Interview Survey as smoking exposure percentage, the daily smoking percentage was calculated using numbers of respondents with cumulative exposure over 100 cigarettes and daily smoking action within 30 days as the denominator, and the total numbers of finished survey as the numerator. National Health Interview Survey, https://www.hpa.gov.tw/Pages/Detail.aspx?nodeid=1077&pid=6198.

## Supplementary Information

Supplementary Information.

## Data Availability

Data are available from the National Health Insurance Research Database (NHIRD) published by Taiwan National Health Insurance (NHI) Bureau. Due to legal restrictions imposed by the government of Taiwan in relation to the “Personal Information Protection Act”, data cannot be made publicly available. Requests for data can be sent as a formal proposal to the NHIRD (http://nhird.nhri.org.tw).
