# Supplementary material for: Evaluating the association of osteoporosis with inhaled corticosteroid use in chronic obstructive pulmonary disease in Taiwan
Source: Sci Rep. 2021 Jan 12;11:724. doi: 10.1038/s41598-020-80815-y (PMC7804267; doi:10.1038/s41598-020-80815-y)
Supplement: Supplementary file 1 — Supplementary Information. [file 41598_2020_80815_MOESM1_ESM.docx]

**Evaluating the Association of Osteoporosis with Inhaled Corticosteroid Use in Chronic Obstructive Pulmonary Disease in Taiwan**

Kai-Lin Chiu, MS^1^; Chun-Chen Lee, MS^2*^, Chung-Yu Chen, PhD^1,2,3,4*^

Author Affiliations:

1. Master Program in Clinical Pharmacy, School of Pharmacy, Kaohsiung Medical University, Kaohsiung, Taiwan.

2. Department of Pharmacy, Kaohsiung Medical University Hospital, Kaohsiung, Taiwan.

3. Department of Medical Research, Kaohsiung Medical University Hospital, Kaohsiung, Taiwan.

4. Center for Big Data Research, Kaohsiung Medical University

*Corresponding author: Chung-Yu Chen

Master Program in Clinical Pharmacy, School of Pharmacy, Kaohsiung Medical University, Kaohsiung, Taiwan

No. 100, Shihcyuan 1st Rd., Sanmin District, Kaohsiung City 80708, Taiwan (R.O.C.)

Tel: 886-7-3121101 ext 2375

Fax: 886-7-3210683

E-mail: jk2975525@hotmail.com

*Corresponding author: Chun-Chen Lee

Department of Pharmacy, Kaohsiung Medical University Hospital, Taiwan

No. 100, Shihcyuan 1st Rd., Sanmin District, Kaohsiung City 80708, Taiwan (R.O.C.)

Tel: 886-7-3121101 ext 6410

E-mail: Lee800296@gmail.com

eTable 1: The definition of baseline characteristics, comorbidities and comedications.

eTable 2: Multivariate conditional logistic regression analysis for variable related to the risk of osteoporosis.

eTable 3. Drug used to define moderate COPD exacerbation.

eFigure 1. The time frame of ICS exposure design.

eTable 1: The definition of baseline characteristics, comorbidities and comedications.

| **Baseline Characteristics** | **Definition** |
| --- | --- |
| **Insurance premium (TWD)** | The level of premium is based on the government announcements. |
| 15,360 | From 2003/01/01 to 2007/06/21 |
| 17,280 | From 2007/06/22 to 2010/12/31 |
| 17,880 | From 2011/01/01 to 2012/12/31 |
| 18,780 | From 2012/01/01 to 2013/03/31 |
| 19,047 | From 2013/04/01 to 2014/06/30 |
| 19,273 | From 2014/07/01 to 2015/06/30 |
| 20,008 | From 2015/07/01 to 2016/12/31 |
| **Comorbidity** | **ICD-9 CM / ICD-10 CM** |
| Asthma | 493 / J452, J453, J454, J459 |
| Dyslipidemia | 272 / E75, E77-E78 |
| Hypertension | 401-405 / I10-I15 |
| Diabetes Mellitus | 250 / E10-E14 |
| Chronic kidney disease | 585 / N184, N185, N186, N189 |
| Chronic liver disease | 070.22, 070.23, 070.32, 070.33, 070.54, 070.59, 070.6, 070.9, 456.0, 456.1, 456.2, 570, 571, 572.2, 572.3, 572.4, 572.8, 573.3, 573.4, 573.8, 573.9, V42.7 / K70, K73, K74 |
| Hyperthyroidism | 242 / E05 |
| Malignancy | 140-239 / C00 – C97, D00 – D49 |
| Acute bronchitis | 466.0 / J20.8, J20.9 |
| Pneumonia | 480, 481, 482, 483, 484, 485, 486 / J12, J13, J14, J15, J16, J17, J18, B25, B44, A221, A3791 |
| Rheumatoid arthritis | 714 / M05 |
| Alcohol-related disease | 291, 303, 305.00, 305.01, 305.02, 305.03 / F10, K70, R78, Z658 |
| Chronic kidney disease | 585 / N184, N185, N186, N189 |
| Dementia | 290.0, 290.1, 290.2, 290.3, 290.4, 294.1 / F05, F03.90, F01.50, F01.51, F02.80, F02.81 |
| Alzheimer's disease | 331.0 / G30.0, G30.1, G30.8, G30.9 |
| Depression | 296.2, 296.3, 300.4, 311 / F32, F34.1 |
| Growth hormone Deficiency | 253.3 / E23.0 |
| Cushing's syndrome | 255 / E24 |
| Hyperparathyroidism | 252.0 / E21 |
| Hypogonadism | 257.2 / E29.1 |
| Hypophosphatasia | 275.3 / E83.3 |
| Porphyria | 277.1 / E80.1 E80.2 |
| Fracture | 805 - 829 / S12, S22, S32, S42, S49, S52, S59, S62, S72, S79, S82, S89, S92, T148, T07 |
| **ASCVD** | |
| Coronary artery disease | 410-414, 429.2 / I20-I25 |
| Peripheral vascular disease | 440-448 / I70, M30, M31 |
| Ischemic stroke/ Transient ischemic attack | 433-437/ G45, G46, I63-I68 |
| Hemorrhagic stroke | 430-432 / I60-I62 |
| Heart failure | 398.91, 402.01, 402.11, 402.91, 404.01, 404.03, 404.11, 404.13, 404.91, 404.93, 425.4, 425.7, 425.8, 425.9, 428 / I50 |
| Left ventricular hypertrophy | 429.3 / I51.7 |
| Atrial Fibrillation | 427, 426 / I440-I447, I450-I459, I462, I468-I472, I479-I481, I490-I495, I498-I499 |
| **Co-medication** | |
| Medication for COPD | |
| LABA | salmeterol, formoterol, indacaterol, olodaterol |
| LABA/ICS | almeterol and fluticasone, formoterol and budesonide, vilanterol and fluticasone |
| LAMA | tiotropium, glycopyrronium, umeclidinium |
| LABA/LAMA | vilanterol and umeclidinium, indacaterol and glycopyrronium, olodaterol and tiotropium |
| SABA | salbutamol, terbutaline, fenoterol, procaterol |
| SAMA | ipratropium |
| SABA/SAMA | fenoterol and ipratropium, salbutamol and ipratropium |
| Systemic beta-2-adrenoreceptor agonists | salbutamol, terbutaline, fenoterol, procaterol, hexoprenaline, tretoquinol, bambuterol, clenbuterol |
| ICS | beclometasone, budesonide, fluticasone, ciclesonide |
| LABA/LAMA/ICS | umeclidinium and vilanterol and fluticasone |
| Methyl-xanthines | diprophylline, choline, theophyllinate, proxyphylline |
| Antibiotic | amikacin, amoxicillin, ampicillin, azithromycin, bacampicillin, benzylpenicillin, cefaclor, cefazedone, cefazolin, cefepime, cefoperazone, cefotaxime, cefotetan, cefotiam, cefoxitin, cefpirome, cefpodoxime, cefradine, ceftazidime, ceftibuten, ceftizoxime, ceftriaxone, cefuroxime, ciprofloxacin, clarithromycin, clindamycin, doxycycline, ertapenem, erythromycin, flucloxacillin, gemifloxacin, gentamicin, imipenem, isepamicin, latamoxef, levofloxacin, linezolid, meropenem, metampicillin, minocycline, moxifloxacin, ofloxacin, oxacillin, penicillin, piperacillin, sparfloxacin, teicoplanin, telithromycin, tetracycline, ticarcillin, tigecycline, trimethoprim and sulfamethoxazole, tobramycin, vancomycin |
| Corticosteroid | betamethasone, dexamethasone, fluocortolone, methylprednisolone, paramethasone, prednisolone, triamcinolone, hydrocortisone, cortisone |
| selective serotonin reuptake inhibitors (SSRI) | fluoxetine, citalopram, paroxetine, sertraline, fluvoxamine, escitalopram |
| anti-epileptic drugs (AED) | carbamazepine, clonazepam, diazepam, gabapentin, dipropylacetamide, divalproex, lacosamide, lamotrigine, levetiracetam, oxcarbazepine, pregabalin, perampanel, phenobarbital, phenytoin, primidone, rufinamide, tiagabine , topiramate, valproate, valproic acid, vigabatrin, zonisamide, alprazolam, bromazepam, brotizolam, chlordiazepoxide, clobazam, estazolam, fludiazepam, flunitrazepam, flurazepam, lorazepam, lormetazepam, medazepam, midazolam, nitrazepam, nordazepam, oxazepam, clorazepate, prazepamm, temazepam, triazolam |
| chemotherapy | anagrelide, aclarubicin, afatinib, alectinib, anagrelide, arsenic trioxide, axitinib, asparaginase, azacitidine, bendamustine, bevacizumab, bleomycin, bortezomib, brentuximab vedotin, busulfan, capecitabine, carboplatin, carboquone, carmustine, ceritinib, cetuximab, chlorambucil, cisplatin, cladribine, clofarabine, crizotinib, cyclophosphamide, cytarabine, dacarbazine, dactinomycin, dasatinib, daunorubicin, docetaxel, doxorubicin, epirubicin, eribulin mesylate, erlotinib, estramustine, etoposide, everolimus, fludarabine, fluorouracil, gefitinib, gemcitabine, hydroxyurea, hydroxyurea, ibrutinib, idarubicin, ifosfamide, imatinib, irinotecan, krestin, lapatinib, lenvatinib, lomustine, melphalan, mercaptopurine, methotrexate, mitomycin c, mitotane, mitoxantrone, nilotinib, nimustine, nintedanib, oxaliplatin, paclitaxel, panitumumab, pazopanib, pemetrexed, pralatrexate, procarbazine, regorafenib, rituximab, ruxolitinib, sorafenib, sunitinib, thioguanine, tretinoin, triethylene , hiophosphoramide, tegafur, temozolomide, temsirolimus, thioguanine, topotecan, trastuzumab, tretinoin, triethylene , thiophosphoramide, trifluridine-tipiracil, uracil-tegafur, vemurafenib, vinblastine, vincristine, vinorelbine, blinatumomab |
| gonadotropin-releasing hormone agents (GRHA) | buserelin, leuprolide, goserelin, triptorelin |
| lithium (Li) | lithium |
| proton pump inhibitors (PPI) | dexlansoprazole, rabeprazole, esomeprazole, lansoprazole, omeprazole, pantoprazole, |
| aromatase inhibitor (AI) | aminoglutethimide, anastrozole, exemestane, letrozole |
| aluminium (AL) | aluminium |
| thiazolidinedione (TZD) | rosiglitazone, pioglitazone |
| thyroid hormone (T4 / T3) | levothyroxine, thyroid, liothyronine |
| androgen deprivation therapy (ADT) | leuprolide, goserelin, buserelin, triptorelin, degarelix, airaterone aetate |
| immunosuppressants | cyclosporine, tacrolimus |
| Anticoagulant drugs |  |
| heparin | heparin |
| warfarin | phenindione, warfarin |
| LMWH | dalteparin, enoxaparin, nadroparin, tinzaparin |

eTable 2: Multivariate conditional logistic regression analysis for variable related to the risk of osteoporosis.

| **3 years** | **Crude** | | | | **Model 1** | | | | **Model 2** | | | |
| --- | --- | --- | --- | --- | --- | --- | --- | --- | --- | --- | --- | --- |
| Variables | **cOR (95% CI)** | | | **p-value** | **aOR (95% CI)** | | | **p-value** | **aOR (95% CI)** | | | **p-value** |
| **ICS use** | 1.056 (1.028, 1.086) | | | <.0001 | 1.053 (1.020, 1.087) | | | 0.0013 | 1.048 (1.015, 1.081) | | | 0.0038 |
| **Smoking** | 1.006 (1.004, 1.008) | | | <.0001 | 1.004 (1.002, 1.006) | | | <.0001 | 1.004 (1.002, 1.006) | | | <.0001 |
| **Age group** |  |  |  |  |  |  |  |  |  |  |  |  |
| 40≤age<50 | 1 (reference) | | |  | 1 (reference) | | |  | 1 (reference) | | |  |
| 50≤age<60 | 0.928 (0.894, 0.962) | | | <.0001 | 0.924 (0.890, 0.959) | | | <.0001 | 0.927 (0.893, 0.962) | | | <.0001 |
| 60≤age<70 | 0.910 (0.879, 0.942) | | | <.0001 | 0.907 (0.875, 0.940) | | | <.0001 | 0.905 (0.873, 0.938) | | | <.0001 |
| 70≤age<80 | 0.953 (0.921, 0.986) | | | 0.0051 | 0.968 (0.934, 1.004) | | | 0.0834 | 0.966 (0.931, 1.002) | | | 0.0615 |
| 80≤age | 0.929 (0.892, 0.967) | | | 0.0003 | 0.975 (0.933, 1.018) | | | 0.2528 | 0.972 (0.931, 1.015) | | | 0.2049 |
| **Male vs Female** | ¶ |  | ¶ |  | ¶ |  | ¶ |  | ¶ |  |  |  |
| **Insurance premium** |  |  |  |  |  |  |  |  |  |  |  |  |
| ≤monthly minimum wage | 1 (reference) | | |  | 1 (reference) | | |  | 1 (reference) | | |  |
| >monthly minimum wage | 1.063 (1.040, 1.086) | | | <.0001 | 1.056 (1.032, 1.081) | | | <.0001 | 1.052 (1.028, 1.077) | | | <.0001 |
| **Urbanization level** |  |  |  |  |  |  |  |  |  |  |  |  |
| Urban | 1 (reference) | | |  | 1 (reference) | | |  | 1 (reference) | | |  |
| Suburban | 1.068 (1.047, 1.090) | | | <.0001 | 1.057 (1.036, 1.079) | | | <.0001 | 1.058 (1.036, 1.079) | | | <.0001 |
| Rural | 1.111 (1.077, 1.146) | | | <.0001 | 1.086 (1.052, 1.121) | | | <.0001 | 1.087 (1.053, 1.122) | | | <.0001 |
| **Comorbidity** |  |  |  |  |  |  |  |  |  |  |  |  |
| Asthma | 1.008 (0.981, 1.035) | | | 0.573 | 0.931 (0.903, 0.960) | | | <.0001 | 0.927 (0.899, 0.956) | | | <.0001 |
| Dyslipidemia | 0.738 (0.690, 0.790) | | | <.0001 | 0.998 (0.975, 1.021) | | | 0.8696 | 0.998 (0.975, 1.021) | | | 0.8645 |
| Hypertension | 1.014 (0.992, 1.036) | | | 0.2066 | 0.953 (0.933, 0.973) | | | <.0001 | 0.953 (0.933, 0.973) | | | <.0001 |
| Diabetes Mellitus | 0.993 (0.973, 1.012) | | | 0.4552 | 1.000 (0.978, 1.022) | | | 0.9737 | 1.002 (0.980, 1.025) | | | 0.8454 |
| Chronic kidney disease | ¶ |  | ¶ |  | ¶ |  | ¶ |  | ¶ |  |  |  |
| Chronic liver disease | 0.994 (0.967, 1.022) | | | 0.6814 | 0.949 (0.922, 0.977) | | | 0.0004 | 0.950 (0.923, 0.977) | | | 0.0004 |
| Malignancy | 0.998 (0.974, 1.022) | | | 0.8446 | 1.021 (0.995, 1.048) | | | 0.1147 | 1.025 (0.999, 1.052) | | | 0.0634 |
| Pneumonia | 0.812 (0.791, 0.834) | | | <.0001 | 0.713 (0.692, 0.735) | | | <.0001 | 0.715 (0.694, 0.736) | | | <.0001 |
| Alcohol-related disease | 2.683 (2.367, 3.040) | | | <.0001 | 2.490 (2.192, 2.829) | | | <.0001 | 2.499 (2.200, 2.840) | | | <.0001 |
| Renal failure | 1.001 (0.946, 1.059) | | | 0.9769 | 0.987 (0.932, 1.045) | | | 0.6479 | 0.988 (0.933, 1.046) | | | 0.6729 |
| Dementia | 1.103 (1.066, 1.143) | | | <.0001 | 1.061 (1.021, 1.101) | | | 0.0022 | 1.067 (1.027, 1.108) | | | 0.0008 |
| Alzheimer's disease | 1.434 (1.286, 1.599) | | | <.0001 | 1.382 (1.235, 1.546) | | | <.0001 | 1.386 (1.239, 1.550) | | | <.0001 |
| Depression | 1.500 (1.444, 1.559) | | | <.0001 | 1.247 (1.192, 1.304) | | | <.0001 | 1.242 (1.188, 1.299) | | | <.0001 |
| ASCVD **^a^** | 1.069 (1.048, 1.089) | | | <.0001 | 1.055 (1.033, 1.078) | | | <.0001 | 1.049 (1.028, 1.072) | | | <.0001 |

eTable 2: Multivariate conditional logistic regression analysis for variable related to the risk of osteoporosis. (continued)

| **3 years** | **Crude** | | | | **Model 1** | | | | **Model 2** | | | |
| --- | --- | --- | --- | --- | --- | --- | --- | --- | --- | --- | --- | --- |
| Variables | **cOR (95% CI)** | | | **p-value** | **aOR (95% CI)** | | | **p-value** | **aOR (95% CI)** | | | **p-value** |
| **Co-medication** |  |  |  |  |  |  |  |  |  |  |  |  |
| oral corticosteroid * | 1.007 (0.988, 1.027) | | | 0.4719 | 1.379 (1.351, 1.408) | | | <.0001 | 1.382 (1.353, 1.411) | | | <.0001 |
| SSRI | 1.577 (1.503, 1.653) | | | <.0001 | 1.271 (1.203, 1.342) | | | <.0001 | 1.270 (1.202, 1.341) | | | <.0001 |
| AED | 1.223 (1.199, 1.247) | | | <.0001 | 1.159 (1.134, 1.185) | | | <.0001 | 1.164 (1.139, 1.190) | | | <.0001 |
| Chemotherapy | 0.791 (0.738, 0.848) | | | <.0001 | 0.786 (0.730, 0.847) | | | <.0001 | 0.812 (0.753, 0.875) | | | <.0001 |
| GnRH agonist | 1.427 (1.210, 1.683) | | | <.0001 | 1.161 (0.201, 6.707) | | | 0.8673 | 1.161 (0.198, 6.824) | | | 0.8686 |
| Aromatase inhibitors | 1.028 (0.828, 1.275) | | | 0.8031 | 1.013 (0.814, 1.262) | | | 0.9046 | 1.026 (0.824, 1.277) | | | 0.8206 |
| Lithium | 1.820 (1.357, 2.440) | | | <.0001 | 1.215 (0.892, 1.655) | | | 0.2163 | 1.211 (0.889, 1.651) | | | 0.224 |
| PPI | 0.967 (0.938, 0.997) | | | 0.0308 | 0.912 (0.883, 0.942) | | | <.0001 | 0.925 (0.895, 0.955) | | | <.0001 |
| TZD | 1.190 (1.086, 1.304) | | | 0.0002 | 1.181 (1.075, 1.297) | | | 0.0005 | 1.180 (1.074, 1.296) | | | 0.0005 |
| Thyroid hormone | 0.869 (0.804, 0.939) | | | 0.0004 | 0.860 (0.796, 0.930) | | | 0.0002 | 0.866 (0.801, 0.937) | | | 0.0003 |
| Immunosuppressants | 1.204 (0.907, 1.598) | | | 0.2 | 1.071 (0.800, 1.432) | | | 0.6461 | 1.077 (0.804, 1.443) | | | 0.6183 |
| ADT | 1.435 (1.218, 1.690) | | | <.0001 | 1.182 (0.206, 6.773) | | | 0.8512 | 1.182 (0.203, 6.891) | | | 0.8526 |
| Aluminum | 1.182 (1.153, 1.212) | | | <.0001 | 1.120 (1.092, 1.150) | | | <.0001 | 1.124 (1.095, 1.153) | | | <.0001 |
| Heparin | 0.627 (0.567, 0.692) | | | <.0001 | - | | |  | 0.641 (0.578, 0.711) | | | <.0001 |
| LMWH | 0.607 (0.487, 0.758) | | | <.0001 | - | | |  | 1.034 (0.968, 1.105) | | | 0.3178 |
| Warfarin | 1.035 (0.970, 1.104) | | | 0.3007 | - | | |  | 0.767 (0.615, 0.956) | | | 0.0182 |
| Anticoagulant **^b^** | 0.878 (0.831, 0.927) | | | <.0001 | 0.867 (0.819, 0.917) | | | <.0001 | - | | |  |
| **No. of** **COPD exacerbations** |  |  |  |  |  |  |  |  |  |  |  |  |
| Moderate exacerbations |  | | |  |  | | |  |  | | |  |
| 0 | 1 (reference) | | |  | 1 (reference) | | |  | 1 (reference) | | |  |
| 1 | 1.021 (0.988, 1.055) | | | 0.2174 | 1.018 (0.984, 1.054) | | | 0.309 | 1.017 (0.983, 1.053) | | | 0.3332 |
| ≥2 | 1.010 (0.979, 1.043) | | | 0.5281 | 1.011 (0.978, 1.046) | | | 0.5183 | 1.010 (0.977, 1.045) | | | 0.5487 |
| Severe exacerbations |  |  |  |  |  |  |  |  |  |  |  |  |
| 0 | 1 (reference) | | |  | 1 (reference) | | |  | 1 (reference) | | |  |
| 1 | 0.977 (0.938, 1.017) | | | 0.2516 | 0.976 (0.936, 1.018) | | | 0.2651 | 0.978 (0.937, 1.020) | | | 0.3027 |
| ≥2 | 0.996 (0.918, 1.081) | | | 0.9303 | 1.024 (0.940, 1.116) | | | 0.5811 | 1.026 (0.942, 1.118) | | | 0.5516 |

eTable 2: Multivariate conditional logistic regression analysis for variable related to the risk of osteoporosis. (continued)

| **3 years** | **Crude** | | | | **Model 3** | | | | **Model 4** | | | |
| --- | --- | --- | --- | --- | --- | --- | --- | --- | --- | --- | --- | --- |
| Variables | **cOR (95% CI)** | | | **p-value** | **aOR (95% CI)** | | | **p-value** | **aOR (95% CI)** | | | **p-value** |
| **ICS use** | 1.056 (1.028, 1.086) | | | <.0001 | 1.047 (1.015, 1.081) | | | 0.0041 | 1.050 (1.017, 1.083) | | | 0.0025 |
| **Smoking** | 1.006 (1.004, 1.008) | | | <.0001 | 1.004 (1.002, 1.006) | | | <.0001 | 1.004 (1.002, 1.006) | | | <.0001 |
| **Age group** |  |  |  |  |  |  |  |  |  |  |  |  |
| 40≤age<50 | 1 (reference) | | |  | 1 (reference) | | |  | 1 (reference) | | |  |
| 50≤age<60 | 0.928 (0.894, 0.962) | | | <.0001 | 0.928 (0.894, 0.963) | | | <.0001 | 0.928 (0.894, 0.963) | | | <.0001 |
| 60≤age<70 | 0.910 (0.879, 0.942) | | | <.0001 | 0.906 (0.874, 0.940) | | | <.0001 | 0.907 (0.875, 0.940) | | | <.0001 |
| 70≤age<80 | 0.953 (0.921, 0.986) | | | 0.0051 | 0.969 (0.934, 1.005) | | | 0.0859 | 0.969 (0.934, 1.004) | | | 0.0844 |
| 80≤age | 0.929 (0.892, 0.967) | | | 0.0003 | 0.974 (0.933, 1.018) | | | 0.2406 | 0.976 (0.935, 1.019) | | | 0.2724 |
| **Male vs Female** | ¶ |  |  |  | ¶ |  |  |  | ** |  |  |  |
| **Insurance premium** |  |  |  |  |  |  |  |  |  |  |  |  |
| ≤monthly minimum wage | 1 (reference) | | |  | 1 (reference) | | |  | 1 (reference) | | |  |
| >monthly minimum wage | 1.063 (1.040, 1.086) | | | <.0001 | 1.054 (1.030,1.079) | | | <.0001 | 1.054 (1.030, 1.079) | | | <.0001 |
| **Urbanization level** |  |  |  |  |  |  |  |  |  |  |  |  |
| Urban | 1 (reference) | | |  | 1 (reference) | | |  | 1 (reference) | | |  |
| Suburban | 1.068 (1.047, 1.090) | | | <.0001 | 1.058 (1.036, 1.080) | | | <.0001 | 1.058 (1.037, 1.080) | | | <.0001 |
| Rural | 1.111 (1.077, 1.146) | | | <.0001 | 1.086 (1.052, 1.121) | | | <.0001 | 1.086 (1.052, 1.121) | | | <.0001 |
| **Comorbidity** |  |  |  |  |  |  |  |  |  |  |  |  |
| Asthma | 1.008 (0.981, 1.035) | | | 0.573 | 0.929 (0.900, 0.957) | | | <.0001 | 0.929 (0.901, 0.958) | | | <.0001 |
| Dyslipidemia | 0.738 (0.690, 0.790) | | | <.0001 | 0.999 (0.976, 1.022) | | | 0.9036 | ** | | |  |
| Hypertension | 1.014 (0.992, 1.036) | | | 0.2066 | 0.953 (0.932, 0.973) | | | <.0001 | 0.952 (0.932, 0.972) | | | <.0001 |
| Diabetes Mellitus | 0.993 (0.973, 1.012) | | | 0.4552 | 1.001 (0.979, 1.023) | | | 0.9386 | ** | | |  |
| Chronic kidney disease | ¶ |  | ¶ |  |  | | |  | ** | | |  |
| Chronic liver disease | 0.994 (0.967, 1.022) | | | 0.6814 | 0.951 (0.924, 0.978) | | | 0.0006 | 0.954 (0.927, 0.981) | | | 0.0011 |
| Malignancy | 0.998 (0.974, 1.022) | | | 0.8446 | 1.025 (0.999, 1.052) | | | 0.0615 | ** | | |  |
| Pneumonia | 0.812 (0.791, 0.834) | | | <.0001 | 0.712 (0.691, 0.734) | | | <.0001 | 0.713 (0.693, 0.735) | | | <.0001 |
| Alcohol-related disease | 2.683 (2.367, 3.040) | | | <.0001 | 2.472 (2.176, 2.808) | | | <.0001 | 2.452 (2.158, 2.785) | | | <.0001 |
| Renal failure | 1.001 (0.946, 1.059) | | | 0.9769 | 0.987 (0.932, 1.045) | | | 0.6489 | ** | | |  |
| Dementia | 1.103 (1.066, 1.143) | | | <.0001 | 1.068 (1.028, 1.109) | | | 0.0006 | 1.065 (1.026, 1.106) | | | 0.0011 |
| Alzheimer's disease | 1.434 (1.286, 1.599) | | | <.0001 | 1.384 (1.237, 1.548) | | | <.0001 | 1.393 (1.245, 1.558) | | | <.0001 |
| Depression | 1.500 (1.444, 1.559) | | | <.0001 | 1.244 (1.190, 1.301) | | | <.0001 | 1.248 (1.193, 1.305) | | | <.0001 |
| ASCVD **^a^** | 1.069 (1.048, 1.089) | | | <.0001 | 1.055 (1.033, 1.078) | | | <.0001 | 1.054 (1.032, 1.076) | | | <.0001 |

eTable 2: Multivariate conditional logistic regression analysis for variable related to the risk of osteoporosis. (continued)

| **3 years** | **Crude** | | | | **Model 3** | | | | **Model 4** | | | |
| --- | --- | --- | --- | --- | --- | --- | --- | --- | --- | --- | --- | --- |
| Variables | **cOR (95% CI)** | | | **p-value** | **aOR (95% CI)** | | | **p-value** | **aOR (95% CI)** | | | **p-value** |
| **Co-medication** |  |  |  |  |  |  |  |  |  |  |  |  |
| oral corticosteroid * | 1.007 (0.988, 1.027) | | | 0.4719 | 1.377 (1.349, 1.406) | | | <.0001 | 1.379 (1.351, 1.408) | | | <.0001 |
| SSRI | 1.577 (1.503, 1.653) | | | <.0001 | 1.270 (1.202, 1.341) | | | <.0001 | 1.269 (1.201, 1.340) | | | <.0001 |
| AED | 1.223 (1.199, 1.247) | | | <.0001 | 1.162 (1.137, 1.187) | | | <.0001 | 1.163 (1.138, 1.188) | | | <.0001 |
| Chemotherapy | 0.791 (0.738, 0.848) | | | <.0001 | 0.787 (0.731, 0.848) | | | <.0001 | 0.797 (0.740, 0.858) | | | <.0001 |
| GnRH agonist | 1.427 (1.210, 1.683) | | | <.0001 | - | | |  | ** | | |  |
| Aromatase inhibitors | 1.028 (0.828, 1.275) | | | 0.8031 | 1.012 (0.813 1.260) | | | 0.9145 | ** | | |  |
| Lithium | 1.820 (1.357, 2.440) | | | <.0001 | 1.201 (0.882, 1.637) | | | 0.2449 | ** | | |  |
| PPI | 0.967 (0.938, 0.997) | | | 0.0308 | 0.914 (0.885, 0.944) | | | <.0001 | 0.916 (0.887, 0.946) | | | <.0001 |
| TZD | 1.190 (1.086, 1.304) | | | 0.0002 | 1.183 (1.077, 1.299) | | | 0.0004 | 1.192 (1.087, 1.307) | | | 0.0002 |
| Thyroid hormone | 0.869 (0.804, 0.939) | | | 0.0004 | 0.860 (0.796, 0.930) | | | 0.0002 | 0.857 (0.793, 0.927) | | | 0.0001 |
| Immunosuppressants | 1.204 (0.907, 1.598) | | | 0.2 | 1.066 (0.797, 1.427) | | | 0.6655 | ** | | |  |
| ADT | 1.435 (1.218, 1.690) | | | <.0001 | 1.324 (1.120, 1.566) | | | 0.001 | 1.322 (1.118, 1.562) | | | 0.0011 |
| Aluminum | 1.182 (1.153, 1.212) | | | <.0001 | 1.122 (1.094, 1.152) | | | <.0001 | 1.121 (1.092, 1.150) | | | <.0001 |
| Heparin | 0.627 (0.567, 0.692) | | | <.0001 | - | | |  | - | | |  |
| LMWH | 0.607 (0.487, 0.758) | | | <.0001 | - | | |  | - | | |  |
| Warfarin | 1.035 (0.970, 1.104) | | | 0.3007 | - | | |  | - | | |  |
| Anticoagulant **^b^** | 0.878 (0.831, 0.927) | | | <.0001 | 0.865 (0.817, 0.915) | | | <.0001 | 0.866 (0.818, 0.916) | | | <.0001 |
| **No. of** **COPD exacerbations** |  |  |  |  |  |  |  |  |  | | |  |
| Moderate exacerbations |  | | |  |  | | |  |  | | |  |
| 0 | 1 (reference) | | |  | 1 (reference) | | |  | ** | | |  |
| 1 | 1.021 (0.988, 1.055) | | | 0.2174 | 1.017 (0.983, 1.053) | | | 0.3255 |  | | |  |
| ≥2 | 1.010 (0.979, 1.043) | | | 0.5281 | 1.011 (0.977, 1.045) | | | 0.5289 |  | | |  |
| Severe exacerbations |  |  |  |  |  |  |  |  |  |  |  |  |
| 0 | 1 (reference) | | |  | 1 (reference) | | |  | ** | | |  |
| 1 | 0.977 (0.938, 1.017) | | | 0.2516 | 0.978 (0.937, 1.020) | | | 0.2948 |  | | |  |
| ≥2 | 0.996 (0.918, 1.081) | | | 0.9303 | 1.024 (0.940, 1.116) | | | 0.5848 |  | | |  |

cOR=crude odds ratio; aOR=adjusted odds ratio; SSRI=Selective serotonin reuptake inhibitors; AED=Antiepileptic drug; PPI= Proton pump inhibitors; TZD=Thiazolidinediones; GnRH agonist=Gonadotropin-releasing hormone agents; ADT=Androgen deprivation therapy; Immunosuppressants=cyclosporine or tacrolimus; LMWH=low-molecular-weight heparin

* The use of oral corticosteroid

a ASCVD=Coronary artery disease + Peripheral vascular disease + Ischemic stroke/ Transient ischemic attack + Hemorrhagic stroke + Heart failure + Left ventricular hypertrophy

b Anticoagulant=Heparin + LMWH + Warfarin

- The factor was not included into adjusted model

** Didn't entry the regression after the stepwise multiple logistic regression

**eTable 3. Drug used to define moderate COPD exacerbation.**

| **Category** | **Drug** |
| --- | --- |
| **Antibiotics** | amikacin, amoxicillin, ampicillin, azithromycin, bacampicillin, benzylpenicillin, cefaclor, cefazedone, cefazolin, cefepime, cefoperazone, cefotaxime, cefotetan, cefotiam, cefoxitin, cefpirome, cefpodoxime, cefradine, ceftazidime, ceftibuten, ceftizoxime, ceftriaxone, cefuroxime, ciprofloxacin, clarithromycin, clindamycin, doxycycline, ertapenem, erythromycin, flucloxacillin, gemifloxacin, gentamicin, imipenem, isepamicin, latamoxef, levofloxacin, linezolid, meropenem, metampicillin, minocycline, moxifloxacin, ofloxacin, oxacillin, penicillin, piperacillin, sparfloxacin, teicoplanin, telithromycin, tetracycline, ticarcillin, tigecycline, trimethoprim and sulfamethoxazole, tobramycin, vancomycin |
| **Corticosteroid** | betamethasone, dexamethasone, fluocortolone, methylprednisolone, paramethasone, prednisolone, triamcinolone, hydrocortisone, cortisone |

**eFigure 1. The time frame of ICS exposure design.**

2-3 year

1-2 year

<1 year

Past use

Recent use

Current use

**Cohort entry date**

**(COPD diagnosis)**

**Index date**

**(osteoporosis diagnosis)**
